# Idiopathic Pulmonary Fibrosis: Epidemiology, Natural History, Phenotypes

**DOI:** 10.3390/medsci6040110

**Published:** 2018-11-29

**Authors:** Jaume Sauleda, Belén Núñez, Ernest Sala, Joan B. Soriano

**Affiliations:** 1 Servei Pneumologia, Hospital Universitari Son Espases, 07010 Palma Mallorca, Spain; belen.nunez@ssib.es (B.N.); ernest.sala@ssib.es (E.S.); 2 Institut de Investigacio Sanitària de les Illes Balears (IdISBa), 07120 Palma Mallorca, Spain; 3 CIBER Enfermedades Respiratorias, Insituto Carlos III, 28029 Madrid, Spain; 4 Instituto de Investigación, Hospital Universitario de la Princesa (IISP), Universidad Autónoma de Madrid, 28006 Madrid, Spain; jbsoriano2@gmail.com

**Keywords:** idiopathic pulmonary fibrosis, prevalence, incidence, evolution, risk factors, comorbidities

## Abstract

Idiopathic pulmonary fibrosis (IPF) is the most common of the idiopathic interstitial pneumonias. It is characterized by a chronic, progressive, fibrotic interstitial lung disease of unknown cause that occurs primarily in older adults. Its prevalence and incidence have appeared to be increasing over the last decades. Despite its unknown nature, several genetic and environmental factors have been associated with IPF. Moreover, its natural history is variable, but could change depending on the currently suggested phenotypes: rapidly progressive IPF, familial, combined pulmonary fibrosis and emphysema, pulmonary hypertension, and that associated with connective tissue diseases. Early recognition and accurate staging are likely to improve outcomes and induce a prompt initiation of antifibrotics therapy. Treatment is expected to be more effective in the early stages of the disease, while developments in treatment aim to improve the current median survival of 3–4 years after diagnosis.

## 1. Introduction

Idiopathic pulmonary fibrosis (IPF) is a chronic, progressive, fibrotic interstitial lung disease of unknown cause, often with characteristic imaging and histological appearances, which occurs primarily in older adults [1]. The characteristic image by thoracic CT (computed tomography) scan shows usual interstitial pneumonia that is microscopically characterized by architectural distortion, interstitial patchy areas, fibroblastic foci, and honeycombing zones, without any inconsistent findings such as inflammation or granulomas [1]. It is considered a rare disease, but recent prevalence and incidence rates appear to be on the rise, probably due to greater awareness, population ageing, male sex, or other factors (see below) [2]. This disease rarely occurs before the age of 50 years [2].

The population burden and costs of IPF have grown due to higher rates of hospital admissions and deaths, the costs of which are secondary to those of disease progression [2]. Its etiology is unknown; however, several risk factors have been postulated such as cigarette smoking, drugs, occupational factors, and infectious or environmental exposures [1]. The natural history of IPF is highly variable and the course of the disease in an individual patient is difficult to predict, as some patients experience rapid lung declines while others progress much more slowly; as such, they may be classified within different groups with different behavior and evolution (phenotypes) [3].

This review is focused on epidemiological aspects, natural history, and phenotypes in IPF.

## 2. Epidemiology

Determining the epidemiology of IPF is a difficult task. Idiopathic pulmonary fibrosis shares problems and limitations with other chronic respiratory diseases, all suffering from blurred definitions, changing coding guidance and expert consensus, high under-diagnosis which often occurs very late in their natural history, as well as the status of rare disease occurrence. Overall, it makes determining and quantifying the distribution of IPF (and its determinants) in time and space a complex issue. We aim to summarize the available evidence on the incidence, mortality and burden of IPF globally and with other levels of granularity, notwithstanding the fact that the results will contain large degree of uncertainty.

### 2.1. Occurrence

IPF accounts for 20% to half of all cases of interstitial lung disease (ILD), and represents the most frequent and severe of the idiopathic interstitial pneumonias (IIPs), a group of ILDs of unknown cause [4]. Idiopathic pulmonary fibrosis is considered a rare disease (occurring in less than 5 per 10,000 person-years), yet its burden is high. In Europe alone, approximately 40,000 new cases are diagnosed each year [5]. Idiopathic pulmonary fibrosis is a clinically heterogeneous disease but its prognosis is overall poor, with a median survival of 3–4 years [6]. Taking all of the above into account, IPF is also an expensive disease, with direct treatment costs of around 25,000 USD/person-year, which is a higher cost in comparison to breast cancer and many other serious conditions [7].

**Figure 1:**
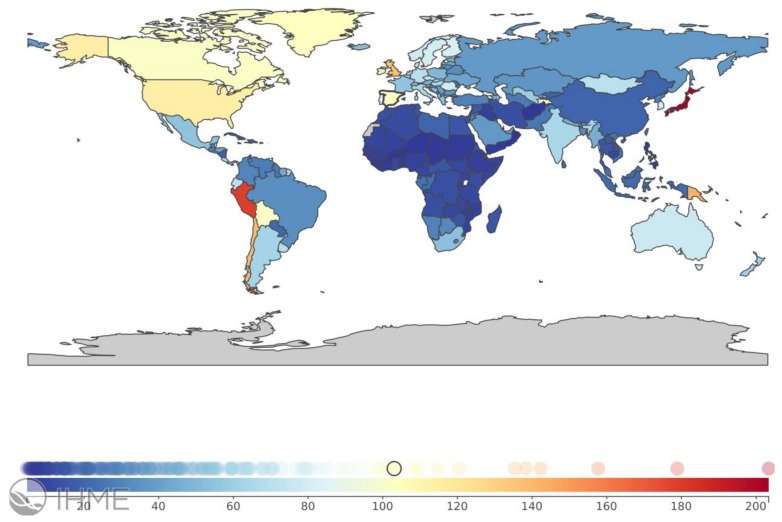
Worldwide burden of interstitial lung diseases and pulmonary sarcoidosis, measured in disability-adjusted life years (DALYs) per 100,000 in 2016, all ages, both sexes, by country. From https://vizhub.healthdata.org/gbd-compare/ (open web site, accessed on 14 July 2018).

The Global Burden of Disease (GBD) [8], an initiative of the Institute of Health Metrics and Evaluation at the University of Washington, WA, USA that works in collaboration with the World Health Organization, does not yet map IPF within its remit. However, IPF is included within the term interstitial lung diseases and pulmonary sarcoidosis (https://vizhub.healthdata.org/gbd-compare). According to the GBD’s tools, there is wide heterogeneity in the distribution of this category of diseases by country, with prevalence ranging 30-fold from 214.5 per 100,000 in Guam to a low of 6.8 per 100,000 in Benin; mortality ranging 150-fold from 10.5 per 100,000 in Japan to a low of 0.064 per 100,000 in Burkina Faso; and the combined estimate of both morbidity and mortality, the so-called disability-adjusted life years (DALYs) ranging more than 60-fold from 173.1 per 100,000 in Guam to a low of 2.7 per 100,000 in Burkina Faso (Figure 1). The already-recognized high IPF burden observed in Japan, some Western European countries, and North America is also accompanied by high rates observed in Peru, Bolivia, and Chile, likely associated with mining, smoking and biomass exposure, genetic factors, or their combination.

### 2.2. Incidence and Prevalence of Idiopathic Pulmonary Fibrosis

Within ILD conditions, IPF accounts for 17–37% of all ILD diagnoses [9]. However, a large variability of prevalence and incidence rates is observed in national and international studies due to a number of limitations. One of the main limitations is the absence of a uniform, consistent definition of IPF in historical series, before the 2000 consensus statement [10]. Also, methods have been used differently for its case ascertainment, from a number of various diagnostic from International Classification of the disease (ICD) codes to death registries and surveys of clinicians with different backgrounds and specialties. The use of different designs means that the results and conclusions of previous studies are not easily or directly comparable.

A study of USA Medicare beneficiaries aged ≥65 years observed IPF rates higher than previously stated (incidence of 93.7 cases per 100,000/year, with a prevalence from 202.2 cases per 100,000 in 2001 to 494.5 cases per 100,000 in 2011) [11]. Within that 10-year period, incidence plateaued while prevalence increased. Other studies suggested an increasing prevalence and a stable or increasing incidence of IPF elsewhere. Most concluded higher prevalence and incidence rates among men and with increasing age, especially after 75 years.

Beyond non-Caucasians, a Japanese study in Hokkaido explored IPF from 2003 to 2007, finding a prevalence of 10 per 100,000/year and an incidence of 2.23 per 100,000/year [12]. They also identified that IPF is more frequent in men and increases with age. Exploring ethnic variability in IPF rates, if any, should be a research theme of interest in the future.

### 2.3. Mortality

Same as with prevalence and incidence rates, mortality statistics are subject to bias. As IPF is an under-recognized and misdiagnosed condition, it is likely to be underestimated at the time of death [13].

Mortality from IPF appears to increase steadily worldwide, even considering the abovementioned overarching underestimation. In 2014, between 28,000 and 65,000 deaths in Europe and between 13,000 and 17,000 deaths in the United States were estimated [13,14].

As with prevalence and incidence, male IPF mortality is higher than in females, increases with age, and appears to be increasingly common [15]. Olson et al. [16] studied IPF mortality from 1992 to 2003 in the USA, reporting an age- and sex-adjusted mortality of 50.8 per 100,000/year, resulting in an increase in deaths by 28.4% for males and 41.3% for mortality during this 12-year period. Why is IPF mortality on the rise? Several factors might be considered, including better screening and case-finding of potential cases. In a USA study exploring causes of death, the most frequent cause was respiratory failure with about 60%, the second most frequent cause was cardiovascular disease (8.5%), and the third was lung cancer (2.9%) [15]. Of interest, the study in Okinawa, Japan reported a very high rate (40%) of acute exacerbation of IPF as cause of death, not seen in other studies [12].

To conclude, IPF fulfils all determinants to be considered a rare disease, but compared with other respiratory diseases, studying IPF epidemiology is not easy for reasons including old studies with heterogeneous methodology and case definitions that have been sequentially modified by experts and consensus. Definitively, IPF prevalence and incidence trends are on the rise. IPF incidence is universally estimated to be around 10 per 100,000 per year, and is uncommon in those younger than 50 years old and more frequent from the sixth decade on. It is more frequent in males than females, and it can be observed in all races and ethnic origins, although in all likelihood with substantial variability. IPF natural history is relentlessly progressive, as is extensively discussed in the next chapter. Yet at the individual patient level, clinical variability is the rule and exacerbations cannot yet be predicted. More and better epidemiology might help us to better determine the burden and unmet needs of IPF, now and in the near future.

## 3. Natural History

### 3.1. Origin

Idiopathic pulmonary fibrosis is a disease of unknown cause, but there is an interaction between genetic and environmental risk factors [17].

Different genetic alterations have been associated with an increased risk of IPF, such as shortened telomeres, oxidative injury, surfactant dysfunction (SFTPC, SFTPA2), proteostatic dysregulation, endoplasmic reticulum stress, mitochondrial dysfunction leading to decreased alveolar epithelial cell proliferation, and the secretion of profibrotic mediators [18]. Mutations in *TERT*, *TERC*, *PARN*, and *RTEL1*—genes involved in the maintenance of telomere length—are associated with an increased risk of IPF [19]. Variations in genes (*DSP*, *AKAP13*, *CTNNA*, and *DPP9*) responsible for cell adhesion, integrity, and mechanic transduction (the generation of electrical signals from a mechanical stimulus) also confer a predisposition to IPF [20,21].

A single-nucleotide polymorphism (rs35705950) in the promoter region of *MUC5B* substantially increases the risk of IPF. *MUC5B* codes for mucin 5B, a glycoprotein required for airway clearance and innate immune responses to bacteria. The rs35705950 minor allele leads to overexpression of mucin 5B in small-airway epithelial cells, a universal finding in patients with IPF (regardless of the *MUC5B* genotype) [22]. Although the mechanism linking mucin 5B overexpression and IPF risk remains unknown, some researchers have hypothesized that aberrant mucociliary clearance may lead to alterations in the lung microbiome and innate immune responses that promote IPF [23,24].

There are also some studies that relate IPF risk, microbiome diversity, host defense pathways (*MUC5B*, *ATP11A*, *TOLLIP*), and disease progression [25,26].

The term epigenetics refers to any process that alters the activity of the gene without changing the sequence of the DNA. Increasing evidence suggests that epigenetic factors contribute to the dysregulation of gene expression in IPF (DNA methylation, histones, and non-coding RNAs modification). Most importantly, risk factors that predispose to IPF—age, gender, cigarette smoke, and genetic variants—all influence epigenetic paths. An association of DNA methylation and histone modifications with the presence of disease and fibro-proliferation has been reported [27]. The miRNAs are short non-coding RNAs (around 20 nucleotides) that regulate gene expression by inhibiting the post-transcriptional expression of the target RNAs.

Let-7d, miR-200, and miR-32, which target the TGFβ pathway and other fibrosis-related pathways, are diminished, while miR-21, which promotes epithelial-mesenchymal transition, is increased in the alveolar cells of lungs of patients with IPF [18].

Clinicians should consider ILD in the differential diagnosis for adults presenting with unexplained exertional dyspnea, chronic dry cough, or crackles on examination. Exertional dyspnea typically progresses over a period of months to years. Often, patients with ILD initially receive a diagnosis of heart failure or chronic obstructive pulmonary disease, suggesting that clinicians frequently fail to consider ILD in patients with dyspnea. In some cases, patients have presented with dyspnea and dry cough up to 5 years before ILD was diagnosed [28].

There are several reasons why there may be a delay in referring patients with suspicion of IPF to a tertiary center. First, patients may wait for months or longer before consulting their general practitioner due to cough and dyspnea in exertion, the most common symptoms of IPF. Secondly, the suspicion of this “rare” disease might be missed by physicians working in primary health care, and recognition of its main clinical sign—crackles in lung auscultation—may be overlooked. Thirdly, a referral to tertiary care may be complicated by shortcomings in the referral letter and the absence of relevant clinical information such as tobacco use, occupational history, and information about previous medication, leading to delays in arranging an appointment and even to inappropriate examinations [29].

Early recognition and accurate diagnosis are likely to improve outcomes through the avoidance of potentially harmful therapies (e.g., glucocorticoids for IPF) and prompt initiation of therapies (antifibrotics) that are effective even in the early stages of disease [2].

### 3.2. Determinants and Risk Factors

The etiology of IPF is unknown, but different risk factors have been identified such as older age, male sex, and cigarette smoking [1,30].

#### 3.2.1. Cigarette Smoking

Cigarette smoking, especially of more than 20 pack-years, is the most strongly associated environmental risk factor. This applies to both familial as well as sporadic IPF. Emphysema and pulmonary fibrosis are both related to smoking and they may even coexist in the same susceptible individuals [31,32]. However, a significant percentage of patients with IPF has never been smokers and animal models of lung injury induction by tobacco smoke activate an emphysematous response of the lung and non-fibrosing; thus, tobacco is still considered an associated factor rather than a clear cause of the disease [33].

#### 3.2.2. Environmental Exposures

Increased risk for IPF has been associated with several environmental exposures (metal dusts, automobile emissions, and wood dust). Activities such as working with livestock and in agriculture have also been associated with IPF [34,35].

#### 3.2.3. Infectious Agents

Several studies have investigated the possible role of chronic viral infection in the etiology of IPF (Epstein-Barr virus, hepatitis C, adenovirus, herpes virus). Currently, definitive conclusions about the role of infection in IPF cannot be made, despite the large number of related studies [36,37]. It is possible that the study of the microbiome in patients with IPF might help us to understand the role of microorganisms in the pathogenesis of IPF.

#### 3.2.4. Gastroesophageal Reflux

Gastroesophageal reflux (GER) probably has a role in the pathogenesis of the IPF and its natural history because predisposition to aspirations or microaspirations of the gastroesophageal content could a constitute factor that triggers damage of the alveolar epithelium, which characterizes IPF. Abnormal GER is frequent in patients with IPF and, usually, is clinically silent. In addition, cases of exacerbation of the disease have been described in the context of aspiration. On the other hand, a decrease in cough has been observed in some cases of IPF in which anti-reflux measures are established. A retrospective study showed that patients with IPF and GER who received treatment for the latter presented lower mortality than patients without treatment [38]; however, recent data do not support these results [39]. Alkaline GER and changes in intrathoracic pressure have been involved in the progression of IPF [40]. However, not all patients with IPF have GER, and the progression of the disease is not clearly associated with the deterioration of the digestive system. The possible role of GER in IPF needs further study.

#### 3.2.5. Autoimmunity

Some autoimmunity phenomena (large lymphocyte aggregates composed of CD3+ T cells and CD20+ B cells as well as the presence of autoantibodies) have been described in patients with IPF, suggesting a breakdown in immunological tolerance [41].

Other risk factors such as diabetes mellitus [42] and obstructive sleep apnea have recently been described [43].

### 3.3. Prognosis

Idiopathic pulmonary fibrosis is a disease with a poor prognosis. Retrospective studies suggest that the median survival time from diagnosis is 3–4 years [1]. In most patients with IPF, the cause of death is IPF-related (i.e., respiratory failure) [44]. In general, older age, male sex, worse dyspnea, and greater lung function abnormality have been associated with weakened prognosis [44].

The natural history of the disease is variable and difficult to predict [45]. Some patients with IPF present a rapid decline in lung function, others progress much more slowly, and some patients show periods of relative stability with acute deteriorations in respiratory function which are unpredictable and often fatal [1]. Many of these events are considered to be acute exacerbations of IPF. Each year, about 10% to 20% of patients with IPF have an acute exacerbation, which is characterized by the worsening of hypoxemic respiratory failure with bilateral ground-glass opacities, consolidation, or both on high-resolution CT imaging that can lead to their death [46]. Exacerbations may be caused by a clinical event such as infection or aspiration, but are frequently idiopathic [46]. Patients with IPF are also at increased risk for venous thromboembolism, lung cancer, and pulmonary hypertension (PH) [47,48,49]. In IPF, an accurate early diagnosis and follow-up might help the clinician to better handle essential decisions such as the effectiveness of antifibrotics (pirfenidone, nintedanib) or the timing of lung transplantation, as well as to empower appropriate end-of-life planning.

Early referral to ILD specialty centers is important for accurate diagnosis and to start therapies that are effective (antifibrotics), even in the early stages of disease, and may be associated with improved outcomes [44].

### 3.4. Interventions: Brief on Treatment and Transplant

The ATS/ERS/JRS/ALAT (American Thoracic Society/European Respiratory Society/Japanese Respiratory Society/Latin American Thoracic Association) guideline for the treatment of IPF includes recommendations for the use of pharmacological and non-pharmacological treatments [10,50]. Two medications, nintedanib and pirfenidone, are safe and effective in the treatment of IPF. Nintedanib, a tyrosine kinase inhibitor that targets growth factor pathways, and pirfenidone, a pleiotropic drug with anti-inflammatory and antifibrotic effects, slow the rate of forced vital capacity (FVC) decline by approximately 50% over the course of one year [51,52] and have shown a reduction in mortality [53,54]. In addition, recent reports show that patients with preserved lung function (FVC > 90% predicted) also benefit from anti-fibrotic treatment [55]. Real-world data from a multi-center, prospective, observational registry support the beneficial effect of anti-fibrotic therapy on survival and transplant-free survival, independent of baseline disease severity, compared to no anti-fibrotic therapy [56]. Although current guidelines recommend the use of antacid therapy to treat IPF [50], there are no data from clinical trials to support this recommendation [2]. Other drugs such as prednisone, azathioprine, *N*-acetylcysteine interferon-γ, endothelin antagonists, and warfarin are ineffective or even harmful in patients with IPF [2].

Pulmonary rehabilitation improves exercise capacity and health-related quality of life [57] while long-term oxygen therapy reduces exertional dyspnea and improves exercise tolerance [50] in patients with IPF; all are recommended in clinical practice guidelines [50].

Lung transplantation can prolong survival and improve lung function and quality of life for selected candidate patients with IPF [58,59]. Whether bilateral is superior to single-lung transplantation in patients with IPF is still unclear, although observational studies and available databases suggest improved outcomes with bilateral transplantation [60]. The referral to a transplant center should be recommended at the time of diagnosis, due to the wide variability in the clinical course of IPF and because the evaluation process and the waiting time for the transplant can be greatly prolonged [61].

## 4. Phenotypes

A phenotype is the outward manifestation of a gene or genes, may involve more than one organ system, and is dynamic, changing over time or in response to environmental factors [62]. However, in the clinical setting the term phenotype is defined as “a single or combination of disease attributes that describe differences between individuals with a disease as they relate to clinically meaningful outcomes” [63]. This definition also raises the question of how much variation within each phenotype is permissible before it can be regarded as a separate entity. This is important because such distinctions make any substantive difference to the patient and their outcomes or to the clinicians and their management of the case.

There is compelling evidence that different phenotypes of IPF can be identified, with different rates of disease progression, including: rapidly progressive, familial, combined emphysema and pulmonary fibrosis, pulmonary hypertension, and that presenting with autoimmune features [3]. Several of these phenotypes are associated with different biomarkers (Table 1).

### 4.1. Rapidly Progressive Idiopathic Pulmonary Fibrosis

The course of patients with IPF is unpredictable and there remains prognosis heterogeneity. On the one hand, there are those patients who survive more than 5 years, usually with higher body mass indices with relatively preserved FVC, TLC, and DLCO [64]. However, on the other hand, some patients experience an accelerated clinical and functional deterioration [3] that could represent another phenotype. Rapid progressors have been associated with low FVC and DLCO as well as with the upregulation of several genes such as those involved in cell motility, myofibroblast differentiation, coagulation, oxidative stress, and development, among them *TLR9*, *CCL18*, antibodies against *HSP70*, and *KL-6* [65]. Lung microbiome has also been related to a faster deterioration of lung function [66]

### 4.2. Familial

Familial pulmonary fibrosis (FPF) is defined by the presence of at least two cases of pulmonary fibrosis within the same family [67]. Although most often these cases are all IPF, this definition does not imply only IPF, because in some families an association of IPF with other ILDs can be observed. It is said that 2–20% of patients with IPF have a first-degree relative with ILD [68]. There are several studies showing that the most important risk for IPF is the presence of a family history [68]. Familial pulmonary fibrosis is more frequent in men and smokers; moreover, it increases with age [68]. The most frequent radiological and pathological pattern is the usual interstitial pneumonia in 80% of cases [68]. The evolution and prognosis are similar to sporadic IPF.

It is believed that the transmission is autosomal dominant. Several genes have been linked with this phenotype: (1) Telomerase complex mutations (*TINF2*, *TERT*, *TERC*) that shorten telomere length; (2) Surfactant protein mutations in specific genes of surfactant proteins called *SFTPA*, *SFTPB*, *SFTPC*, and *SFTPD* [69]. Among these mutations, the most reported in FPF are *SFTPC* ones; (3) Other syndromes with pulmonary fibrosis associated with monogenic disorders that are also associated with extrapulmonary manifestations have been described. The most typical are the Hermansky–Pudlak syndrome (oculocutaneous albinism, predisposition to bleeding) and type I neurofibromatosis (cutaneous neurofibromas, “café au lait” skin lesions, pigmented hamartomas of the iris) [69]; (4) In addition to monogenic Mendelian law, several genetic polymorphisms have been associated with FPF. The best known is *MUC5B*, but other have also been related to IPF, i.e., *TLR9* and *TERT* [69].

Epigenetic changes have also been associated with FPF, such as methylation modifications of certain genes and microRNAs [70].

### 4.3. Combined Pulmonary Fibrosis and Emphysema (CPFE)

Several studies have suggested that IIPs commonly associate with combined pulmonary fibrosis and emphysema (CPFE) and mostly (28–35%) with IPF [71,72]. It is unclear if emphysematous and fibrotic diseases progress independently, or whether one is the consequence of the other [71]. The high prevalence of cigarette smoking in patients with IPF could explain the high prevalence of emphysema; however, the pathophysiology of this syndrome has not been completely identified. There are several hypotheses relating this syndrome to exposure to chemicals, overexpression of TNF-alpha and PDGF-beta, polymorphisms in some genes (*MMP-1* or *SFTPC* genes), and shorter telomeres [67,71].

Combined pulmonary fibrosis and emphysema is most frequent in males older than 65 years, and active or former smokers. It is characterized by severe dyspnea, very impaired transfer capacity for carbon monoxide, preserved lung volumes, and hypoxemia during exercise. Its diagnosis is based upon high-resolution computed tomography (HRCT) by the presence of fibrotic changes more evident in the lower lobes and the emphysematous component more present in the upper lobes [71].

CPFE frequently (about 50%) presents with pulmonary hypertension, conferring worse prognosis than IPF alone, with a median survival time of about 25 months [72].

There are no specific treatment recommendations for CPFE. It might be reasonable to treat both components—bronchodilators to treat the obstruction associated with the emphysematous component and antifibrotics for IPF [71]. Regarding the latter, there are no specific recommendations about a specific antifibrotic, although a post-hoc subgroup analysis of both INPULSIS trials showed that nintedanib slowed disease progression independent of the presence of emphysema at baseline [73].

### 4.4. Pulmonary Hypertension

Pulmonary hypertension (PH) is often found in patients with IPF, and its occurrence increases with IPF severity. Its prevalence remains unclear and estimates vary widely (from 3% to 86%), reflecting the heterogeneous subpopulations of patients studied, as well as the underlying disease severity, disease definition, and different diagnostic measures used [47]. The presence of PH is associated with an increased risk of death, especially if systolic pulmonary arterial pressure (sPAP) by echocardiogram exceeds 50 mmHg [74] or mean pulmonary arterial pressure (mPAP) by right heart catheterization (RHC) is greater than 20 mmHg [75]. The median survival time from IPF diagnosis among patients with PH ranges from approximately 2–3 to 4 years [76].

Echocardiography is the test of choice for the detection of PH, although its accuracy in patients with advanced respiratory disease is low. Definitive diagnosis of PH is established by RHC [77].

At present, there is no specific therapy for PH associated with IPF. Published experience with targeted PH drug therapy is scarce, and so far there is no evidence from randomized controlled trials (RCTs) suggesting that PH drugs improve symptoms or other outcomes in patients with IPF [77]. Hence, until further evidence is available, the use of drugs approved for PH should be avoided in patients with IPF unless they are enrolled in an RCT investigating such therapies [2]. Conversely, patients with suspected PH, in addition to their lung diseases (characterized by mild lung parenchymal abnormalities, symptoms insufficiently explained by lung mechanical disturbances, and a hemodynamic PH phenotype, i.e., severe PH with high Pulmonary vascular resistance (PVR) and low cardiac output (CO)) may be treated according to the recommendations for PH. It is important to keep in mind the potential implications of the co-existing lung disease on symptoms and response to therapy and that the use of specific treatments for PH such as ambrisentan and riociguat are specifically contraindicated in IPF [78]. These patients should be referred to a specialized centre for individualized treatment [77].

### 4.5. Connective Tissue Diseases

Connective tissue diseases (CTD) are a group of diseases characterized by circulating autoantibodies and systemic manifestations considered to be related to autoimmune-mediated organ damage. They include rheumatoid arthritis, systemic sclerosis, poly/dermatomyositis, systemic lupus, Sjögren’s syndrome, and mixed connective tissue disease [79]. Most of them can present ILD, including the usual interstitial pneumonia that is found mainly in rheumatoid arthritis. However, strictly speaking, we should not label such cases as IPF, because the pulmonary fibrosis associated with these diseases is not considered idiopathic. Nevertheless, there exist reports of some patients with ILD and combinations of other clinical, serologic, and/or pulmonary morphologic features which putatively stem from an underlying systemic autoimmune condition, but do not meet current rheumatologic criteria for a characterized CTD, the so-called interstitial pneumonia with autoimmune features (IPAF). The ATS and ERS have proposed IPAF criteria that include several domains (clinical, serological, and morphological) [80]. However, the first approach does not include the usual interstitial pneumonia, an issue that has been raised by some authors who believe that it should be incorporated because the usual interstitial pneumonia is one of the most radiological and pathological patterns in CTD [81]. Adopting IPAF classification means leaving behind the previously accepted terminologies, and allows for the future study of a more uniform cohort. Prospective studies are urgently needed to validate the proposed classification criteria and to determine the natural history and clinical implications of a classification of IPAF [80].

The treatment of IPF in this phenotype should be the same as that of the CTD—mostly systemic steroids and/or immunosuppressive agents [82].

### 4.6. Other Phenotypes

Some authors proposed IPF acute exacerbators as a phenotype “per se”, but patients who develop acute exacerbations can merely be considered as an IPF complication [83]. The different etiologies of acute exacerbations such as occult aspiration, viral infections, or other insults might be the precipitating factors. Therefore, exacerbation could be considered as a complication [83].

Other potential phenotypes might be the radiological phenotypes. However, nowadays some radiological features in IPF are considered to be related to the prognosis rather than to new phenotypes. On the one hand, the more extended interstitial infiltrates and/or honeycombing, the poorer prognosis; on the other hand, an atypical pattern confers a better prognosis [83]. The appearance of the disease as assessed by HRCT may be regarded as a physical feature while radiographic variants probably will be regarded as specific disease phenotypes. In this regard, the presence of traction bronchiectasis and asymmetric disease have been reported to be associated with different outcomes [84].

Pulmonary function tests (PFTs) do not define distinct phenotypes, but certain characteristics can be associated with some phenotypes [83]. For instance, airflow obstruction might be seen in CPFE [71].

## Figures and Tables

**Table 1:** Biomarkers (proteins and genes) associated with specific phenotypes.

Phenotype	Biomarker
Rapidly progressive	TLR9 CCL18 Antibodies against HSP70 KL-6
Familial	Telomerase complex mutations: TINF2, TERT, TERC Surfactant protein mutations: SFTPA, SFTPB, SFTPC, SFTPD
Combined pulmonary fibrosis and emphysema (CPFE)	TNF-alpha and PDGF-beta Genes polymorphisms: *MMP-1* or *SFTPC* genes
